# Clinical Status of Efflux Resistance Mechanisms in Gram-Negative Bacteria

**DOI:** 10.3390/antibiotics10091117

**Published:** 2021-09-16

**Authors:** Anne Davin-Regli, Jean-Marie Pages, Aurélie Ferrand

**Affiliations:** Membranes et Cibles Thérapeutiques-Faculté de Pharmacie, UMR_MD1, U-1261, Aix-Marseille Université, 27 Boulevard Jean Moulin, 13385 Marseille, France; jean-marie.pages@univ-amu.fr (J.-M.P.); ferrand.aurelie@hotmail.fr (A.F.)

**Keywords:** RND efflux pumps, prevalence of efflux resistance mechanisms, hospital acquired infections

## Abstract

Antibiotic efflux is a mechanism that is well-documented in the phenotype of multidrug resistance in bacteria. Efflux is considered as an early facilitating mechanism in the bacterial adaptation face to the concentration of antibiotics at the infectious site, which is involved in the acquirement of complementary efficient mechanisms, such as enzymatic resistance or target mutation. Various efflux pumps have been described in the Gram-negative bacteria most often encountered in infectious diseases and, in healthcare-associated infections. Some are more often involved than others and expel virtually all families of antibiotics and antibacterials. Numerous studies report the contribution of these pumps in resistant strains previously identified from their phenotypes. The authors characterize the pumps involved, the facilitating antibiotics and those mainly concerned by the efflux. However, today no study describes a process for the real-time quantification of efflux in resistant clinical strains. It is currently necessary to have at hospital level a reliable and easy method to quantify the efflux in routine and contribute to a rational choice of antibiotics. This review provides a recent overview of the prevalence of the main efflux pumps observed in clinical practice and provides an idea of the prevalence of this mechanism in the multidrug resistant Gram-negative bacteria. The development of a routine diagnostic tool is now an emergency need for the proper application of current recommendations regarding a rational use of antibiotics.

## 1. Introduction

**Figure 1:**
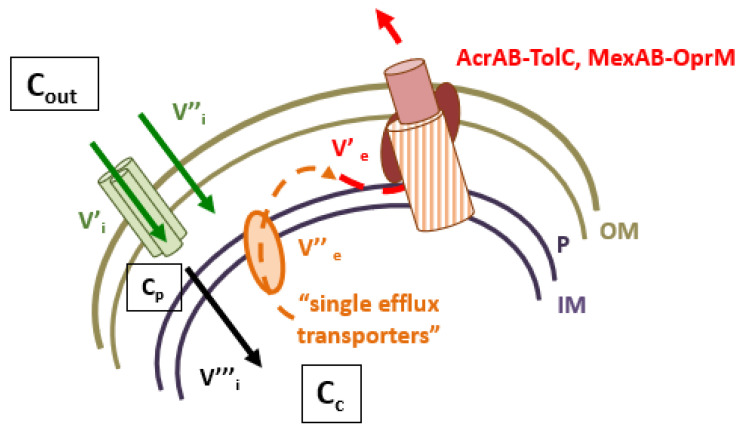
Intracellular concentration of antibiotics and RND efflux pumps. V’_i_ and V’’_i_ represent the influx rate across the outer membrane, V’’’_i_ represents the diffusion rate across the inner membrane. C_out_, C_p_ and C_c_ represent the external, periplasmic and cytoplasmic concentration of antibiotics, respectively. V’’_e_ and V’_e_ represent the efflux rate accross the inner and outer membranes. AcrAB-TolC and MexAB-OprM illustrate the archetype of efflux pumps detected in clinical isolates.

Membrane-associated antibiotic resistance is a key mechanism in Gram-negative bacteria that efficiently controls the intracellular concentration of various drugs [1]. Two complementary processes, membrane impermeability and the expression of efflux pumps, limit the concentration of deleterious compounds inside bacteria by impairing the entry and expelling the internal molecules [1,2,3,4]. Consequently, they play a key role in protecting the bacterial cells against aggressive chemicals, such as antibiotics, disinfectants, conservatives, detergents, etc. In Gram-negative bacteria, the envelope permeability has long been studied, but due to its complex organization and variability, serious gaps in the research remain, regarding the antibiotic translocation and accumulation inside living bacteria. Multidrug efflux pumps active in bacterial cells belong to the ABC, MF, SMR, MATE, PACE and RND superfamilies superfamilies [4,5]. Except ABC transporters using ATP hydrolysis as energy source, the other drug efflux pumps are antiporters, H^+^ or Na^+^, with the active component inserted in IM (Inner/Cytoplasmic Membrane) [1,4]. This component recognizes and captures the drug substrate from the cytoplasm or IM surface and carries the molecule to an outer membrane channel that releases it to external medium. In Gram-negative bacteria, AcrAB-TolC and MexAB-OprM are the archetypes of the tripartite efflux pump complexes reported in clinical strains (Figure 1). The regulation of the expression of the pump, and its 3D organization, have been extensively studied and reported in numerous reviews with three AcrAB-MexAB in IM for three TolC-OprM partners in OM (Outer Membrane) [1,4,5,6,7,8]. Interestingly, these transporters exhibit an important versatility and flexibility of substrates, from bile salts to dyes including antibiotics and detergents with a large range of sizes, chemical structures, and charges [1]. The mechanism of efflux as a protection barrier has been genetically and molecularly studied. The functional structures of efflux complexes, e.g., AcrAB-TolC and MexAB-OprM, have been solved at high resolution providing information regarding the energy requirements, dynamic and selectivity of the transport, which includes a recognition, binding and expel step [1,2]. Today, the role of specific amino acids residues during the transport inside the different AcrB-MexB pockets and the subsequent conformational changes of the efflux pump subunits are described [1,4,5].

Importantly, clinical strains express a basal level of efflux pumps and can overproduce them via mutation in regulators (negative or positive), stimulation by external stresses, or the acquisition of mobile genetic elements coding for additional transport systems [1,5]. A sophisticated regulation cascade can efficiently manage the membrane transporters, porins and efflux components, which are involved in drug transport [5,9].

However, the exact prevalence and contribution of efflux in clinical pathogens are not well defined [1]. This gap is due to a lack of clinical diagnosis assays that impair a rapid and clear identification of active effluxes in hospital microbiology services in contrast to available methods for detecting enzymes or target mutations involved in resistance (inhibitor of β-lactamase, sequence of specific gene, etc.) [1]. Today, the vast majority of clinical studies carried out on efflux resistance use either the ratio of MICs obtained in the absence or presence of efflux inhibitors, the interpretation of the data obtained after the evaluation of the impact of the target mutations or the presence of enzyme barrier, or, more recently, the use of a synthetic substrate of an efflux pump devoid of antibiotic properties (e.g., ethidium bromide or other chemicals). Consequently, it dramatically lacks a real-time approach based on the measurement of the accumulation of an antibiotic correlated with its antibacterial activity to precisely define the role, prevalence, and impact of efflux in clinic.

## 2. Towards an Detection of Efflux Mechanisms

During the last decade, the respective developments of fluorimetry and mass spectrometry have generated several experimental protocols supporting a serious breakthrough in the precise quantification of the intracellular accumulation of drugs inside bacteria. Consequently, the impact of membrane impermeability and active efflux in resistant isolates have been revealed [3,10], allowing for a clear quantification of the role of these membrane-associated mechanisms in the resistance [1,11,12]. The key point is the requirement of the robust internal controls necessary to validate the drug signals and to allow comparisons between various assays involving various bacterial strains [12]. Unfortunately, it seems difficult today to efficiently transfer these research protocols in simple routine tests for hospital diagnosis. This point could be partly solved by using a first screen in a collection of Multi-Drug Resistant (MDR) isolates and determining the accumulation of selected antibiotics used in clinical settings in a second step. The final analyses will be the determination of the existing correlative index between the accumulation level and susceptibility for molecules that have been classified as a substrate of efflux pumps [3]. This aspect is especially important to define the relevance and contribution of efflux in clinical isolates and the rapid adaptation of bacterial cells under antibiotic pressure.

## 3. Genetic Regulation

The expression of the various efflux systems is controlled by an interplay of different regulators and regulation cascades, some of which are major players and other substitutes or alternate pathways [9]. The regulation signal seems to be associated with the internal concentration of toxic chemicals that play a role during the trigger of the cascade-inducing efflux expression [9]. In Gram-negative bacteria, the genetic regulation of pumps is mainly managed by two key methods:

1. Pathway 1: the intervention of a protein-modulating expression of genes by a fixation on their promoters. Regulatory genetic proteins possess α-helix-turn-α-helix (HTH) DNA-binding motifs and can be activators or repressors [13].
2. Pathway 2: the activation of a two-component systems (TCS) which interferes with gene expressions when environmental stresses require a bacterial adaptation [1].

### 3.1. HTH Family Regulators

Positive regulation is managed by the AraC-XylS family, which is well-documented in *Enterobacteriaceae* (MarA, RamA, SoxS, Rob, RarA) or H-NS proteins (histone-like structuring nucleoid protein) (SdiA, FIS, CsrA). In this group, representative repressors are TetR/AcrR/RamR/MexR and the OqxR family and are described in *Enterobacteriaceae* and in *Pseudomonadaceae*. They are repressors of the genetic cascade regulation or of the pumps genes operons [9].

### 3.2. TCS Systems

TCS systems are activated when bacteria sense environmental change such as detrimental hazards to their key physiological activities, and contribute to adaptation/defense. TCS systems (CpxAR, Rcs, BaeSR, PhoPQ, and EnvZ/OmpR) (RocS2-RocA2, ParR-ParS, AmgR-AmgS, CzcR-CzcS and CopR-CopS) can sense the external medium modifications such as pH, osmotic strength, oxidative stress, nutrient starvation, and toxic chemicals, etc. [1].

## 4. Clinical Impact Situation

The basal efflux present in bacterial cells is essentially the first mechanism with membrane impermeability that faces antibiotics in clinical isolates [14,15,16]. The basal activity is not clinically detected in a wild strain, although various stimuli are able to quickly initiate their overexpression and synthesis. Although not detected in routine, the efflux paves the way for the most radical mechanisms of resistance [3]. A sub-inhibitory intracellular concentration of the antibiotic then promotes the development or acquisition of more specific mechanisms of resistance-like enzymatic responses or target mutations [15,16]. For example, several mutations are found in genes, *gyrA* and *parC*, coding the gyrase target and they are frequently associated with efflux resulting in high MIC levels [17]. Most of the studied clinical strains exhibit a specific resistance combined to efflux mechanisms (Table 1). The inhibition of efflux mechanisms obtained in the laboratory for these strains never completely restores susceptibility to the antibiotic despite a significative diminution of MIC. Given the lack of automated techniques available to monitor efflux activity, efflux is not usually sought in epidemiological studies involving samples of clinical strains; nevertheless, some studies have been investigated, particularly with *Pseudomonas aeruginosa* [18]. We review the main results and observations of efflux in a clinical context for the various species most concerned.

### 4.1. Haemophilus influenzae

In *Haemophilus influenzae*, an AcrAB homolog pump system is identified and shares a similar antibiotic efflux profile as AcrAB overexpression generates in *E. coli* [19].

The basal expression of the efflux represents a current mechanism which concerns macrolides and ketolides resistance in most clinical strains [20]. Efflux is frequently found to be associated with mutations in ribosomal proteins resulting in high MICs to these classes of antibiotics [20,21]. Recently, Cherkaoui et al. studied various resistance mechanisms for imipenem heteroresistance in 46 *Haemophilus influenzae* isolates [22]. They concluded that the involvement of the pump AcrAB overexpression caused by a partial deletion in *acrR* led to an overexpression of the AcrAB-TolC efflux complex. For them, efflux was necessary for the development of imipenem heteroresistance [22].

### 4.2. Helicobacter pylori

In *Helicobacter pylori*, several efflux pumps systems were identified: RND pump complexes named HefABC, HefDEF and HefGHI, ABC and MFS-type efflux pump not yet named [23].

From two series of *H. pylori* clinical isolates resistant to clarithromycin which was the key drug in the eradication therapy for *H. pylori*, two TolC homologs, hp0605 and hp1489, were identified as involved in clarithromycin resistance [24]. Moreover, metronidazole resistance was reported in five MDR clinical isolates exposed to varying concentrations of this antibiotic, and the efflux mechanism was observed in this phenotype [24]. Authors reported that metronidazole could stimulate the expression of efflux pumps genes and expression occurred when higher doses of metronidazole were used [25]. Among 12 clinical strains resistant to clarythromycin, the genetic variants of the three RND pumps of the series of Hef were identified [26]. Authors detected specific single-nucleotide substitutions in resistant strains [26].

### 4.3. Campylobacter jejuni

*Campylobacter jejuni* followed by *Campylobacter coli* are the most common bacteria isolated in campylobacteriosis [27]. Macrolides, with erythromycin or fluoroquinolones, are the first line of treatment. The mechanisms of resistance to macrolides and fluoroquinolones are independent and, for macrolides, the resistance mechanisms are also described for other members of this antibiotic class as the ketolide telithromycin [28]. There are two major mechanisms of antibiotic resistance: target mutations and the decrease in the intracellular concentration of antibiotics. The decrease in the intracellular concentration of antibiotics may occur via efflux pump activity, which was described for the first time in 1995 [29].

*CmeABC* is the major efflux complex that can extrude a large panel of molecules [30]. *CmeB* is the inner membrane transporter (energy-active part), *CmeC* is the outer membrane channel protein, and *CmeA* is the periplasmic fusion protein [30,31]. *cmeR,* located in the promoter region of *cmeABC* operon, encodes *CmeR*, a Tet-like regulator, which controls the efflux complex expression. The *CmeR* acts as a local repressor for *CmeABC* [32].

Interactions with other efflux complexes like *CmeDEF* may result in intrinsic resistance to these antibiotics [33]. A resistance-enhancing variant of *CmeABC* called RE-*CmeABC* is described as a “super efflux pump” which confers to *Campylobacter* at a higher resistance level than *CmeABC*. This super efflux pump can extrude more antibiotics than the *CmeABC* and a mutation is found in the promoter region of RE-*cmeABC*. Furthermore, the study of the structural modeling of *CmeB* points to amino acid changes that suggest a tighter affinity of this variant to the drugs, compared to parental *CmeB* conferring a fitness benefit to *Campylobacter* under selection pressure. The spreading of this pump is characterized by a horizontal transfer and a clonal expansion [34].

There are two levels of macrolide resistance in *Campylobacter* spp.: a low level of resistance (LLR) and a high level of resistance (HLR) [28]. Regarding the macrolide susceptibility, the LLR strains have no target mutations and are susceptible to the PAβN (Phe-Arg-β-naphthylamide) efflux pump inhibitor, while the HLR strains show target mutations that cannot be bypassed with PAβN addition [35]. It has been shown that in *Campylobacter* clinical isolates, concerning fluoroquinolones, strains target mutations and efflux pump activity works synergistically. However, the resistance to fluoroquinolones may occur only with a *gyrA* mutation, with or without an overexpression of the *CmeABC* efflux complexes [36,37]. Conversely, the efflux alone requires the contribution of several pumps to generate fluoroquinolones resistance without target mutations in *Campylobacter* [38,39].

### 4.4. Enterobacteriaceae

*Enterobacteriaceae*, which includes over 30 genera and more than 100 species, are common pathogens in the community and in hospital. Some of them are associated with worldwide epidemics, such as *Salmonella*, *Escherichia coli* and *Shigella* sp. Moreover, they are involved in about 40% of hospital acquired infections, half of them associated with *E. coli* and others corresponding to *Klebsiella pneumoniae* and *oxytoca*, *Enterobacter cloacae* and *K. aerogenes, Proteus* *mirabilis* and *Citrobacter flexneri* [9,14,18]. Among them, it must be noted that the *E. coli* AcrAB-TolC represents the archetype of the tripartite RND efflux complex regarding structural organization, genetic regulation, and activity [1,2,3,4,5,6].

#### 4.4.1. *K. pneumoniae*

*K. pneumoniae* comes after *E. coli*, the main enterobacterial species involved in nosocomial infections, with the highest proportion of isolates producing plasmid-mediated extended-spectrum β-lactamases and carbapenemases [40,41,42,43]. Two major RND pumps and their regulatory elements are characterized in *K. pneumoniae*, AcrAB and OqxAB [40,44]. The *oqxAB* operon was originally described on plasmid carried by an *E. coli* strain conferring resistance to chloramphenicol and quinolones [44]. In *K. pneumoniae,* it is associated with the resistance of quinoxalines, quinolones, tigecycline, nitrofurantin and chloramphenicol [45]. The major genetic regulators of these two pumps are RamA and RarA, respectively, together belonging to the AraC-type transcriptional regulators in *K. pneumoniae*, [46,47]. AcrAB is involved in the efflux of various antibiotics including the most recent broad-spectrum antibiotics, such as piperatazocillin or ceftolozane-tazobactam, and OqxAB expels fluoroquinolones, tigecyclin and nitrofurantoin [45]. Concerning the detection of efflux mechanisms and their prevalence in the resistance of clinical strains, Kareem et al. showed that 27 of the 43 selected MDR *K. pneumoniae* clinical strains presented an efflux activity by using an EtBr-agar cartwheel screening method [48]. In 36 MDR clinical isolates in Egypt, *acrAB* genes were found in 82% of tested strains [49]. In 2017, in a Taiwan hospital, 17 MDR *K. pneumoniae* responsible for urinary tract infections were found to have an upregulation and an overexpression of *acrB* and/or *oqxB* in 65% of strains [50]. They were associated with mutations or insertions in OmpK36 porin that altered outer membrane permeability. Elgendy et al. found, in Egyptian hospitals, that 12% of *K. pneumoniae* strains exhibited an overexpression of AcrAB and OqxAB associated with tigecycline resistance [51]. In the same aspect, Sekyere et al. observed that, among isolates from Durban hospitals, half of them presented an efflux mechanism associated with fluoroquinolones resistance [52].

In addition to these two major pumps, other transport proteins systems were described and found involved in clinical resistance. By using transcriptome analysis, Majumdar et al. found that the overexpression of the regulator RarA resulted in the overexpression of genes encoding uncharacterized transport proteins (KPN_03141, SdaCB, and LeuE) [47]. Moreover, Srinivasan et al. first characterized in *K. pneumoniae*, the KpnEF efflux pump [53]. Maurya et al. reported the role of new pumps in *K. pneumoniae* in resistance: for the RND family, KexD (for macrolides and tetracycline); for the SMR family pump, KpnEF, and for the MATE family, KdeA (involved in chloramphenicol, norfloxacin, acriflavine, and ethidium bromide (EtBr) resistance) [54]. Interestingly, KpnEF seemed to be involved in the transport of polysaccharides to the outer layer to form the slimy layer in connection with biofilm formation. The KpnEF pump also mediated resistance to antibiotics such as cefepime, ceftriaxone, colistin, erythromycin, rifampin, tetracycline, streptomycin and seemed to have a significative role in the MDR phenotype [53]. Southern hybridization of the genomes of several MDR strains indicated a 70% occurrence for *kpnEF*. Authors concluded that these supplementary pumps did not contribute much to resistance but provided a synergistic/additive effect in the efflux of β-lactam family.

Lv et al. (2021) found in a clinically isolated pan-resistant *Klebsiella pneumoniae* strain, which was resistant to β-lactams, sulfonamides, bacitracin, tetracycline, aminoglycosides, and chloramphenicol, a combinate involvement of enzymatic mechanisms, and efflux pumps co-intervention [55]. Five types of efflux pump families were identified in clinical strains, including RND pumps (AcrAB, AcrD, MdtABC, and KexD), the ABC superfamily (MacAB), SMR (KpnEF), MATE (KdeA), and the MFS superfamily (EmrAB). Moreover, they identified mutations in the deletion mutations of the regulatory genes, *acrR* and *ramR,* which led to the overexpression of the AcrAB efflux pump [55].

#### 4.4.2. *K. aerogenes*

A range of MDR efflux pumps: AcrAB, AcrD, EmrAB, MacAB, MdtABC, OqxAB, RosAB composed the main resistance efflux mechanisms encoded in *K. aerogenes* [46,56]. The EefABC and AcrAB-TolC complexes of *K. aerogenes* were particularly studied at the functional level [57,58,59,60].

Regarding the prevalence of efflux and its evolution in clinical strains, rare specific studies have been published: Chevalier et al. studied efflux mechanisms in two groups of *K. aerogenes* collected in two different periods (1995 and 2003) and their level of resistance was studied and compared within the 8-year period. A noticeable increase in efflux mechanism expressions is observed in one decade using PAβN as an efflux reporter [61]. They concluded that approximately 40% of MDR clinical strains exhibited an active efflux in 2003. Tran et al. investigated the occurrence of efflux and other mechanisms in 44 *K. aerogenes* and *Klebsiella pneumoniae* clinical isolates [43]. A phenotypic efflux in the presence of PAβN was detected in *K. aerogenes* isolates, more frequently than in *K. pneumoniae* (100 versus 38% of isolates). Gayet et al. explored two MDR strains resistant to broad spectrum β-lactams antibiotics, fluoroquinolones, chloramphenicol, tetracycline, and kanamycin [62]. In the presence of PaβN, the tested *K. aerogenes* strains resulted in up to a fourfold decrease in the chloramphenicol MIC, suggesting a significative efflux mechanism. The OqxAB pump was also investigated for its involvement in *E. cloacae,* as in *K.* *aerogenes* strains, and it was shown that it contributed to a decreased susceptibility to fluoroquinolones [63].

#### 4.4.3. *E. coli*

AcrAB is the major MDR efflux pump in *E. coli*, with a constitutive expression in wild-type strains and a significant overexpression during drug exposure [64,65]. Close homologs of AcrAB among the RND pumps family are AcrD, AcrEF, MdtABC and YhiUV (MdtEF), which play a minor role because their in vitro expression is only triggered after AcrB inactivation [65]. Finally, MdfA transporters belong to the MFS family and are known to be related to quinolone resistance [66]. Most of the efflux pumps are conserved across the different *E. coli* phylogroups [64]. Despite the importance of *E. coli* in a clinical setting, limited studies concern the role of efflux in hospital infection situations. In a Japan study, in 2008, authors observed that among 64 *E. coli* isolates obtained from patients with urinary tract infections in posttreatment, 52 isolates (81.3%) presented an overexpression of the *marA* gene that upregulated *acrAB* [66]. Moreover, 26.6% overexpressed *yhiV (mdtF)* and 34.4% *mdfA*. Authors concluded that there was a correlation between the AcrAB and MdfA efflux systems in resistance to fluoroquinolones [66]. In 2018, 200 non-repetitive *E. coli* strains isolated from urine samples of patients admitted to the Hospital of Wenzhou in China were collected [67]. Strains presented a high resistance rate to ampicillin, fluoroquinolones, gentamicin and 2.5% were found to be resistant to triclosan. Increased RT-qPCR expressions were noted for most of the efflux pumps encoding genes such as *ydcV*, *ydcU*, *ydcS*, *ydcT*, *cysP*, *yihV*, *acrB*, *acrD*, and *mdfA.* ABC transport efflux pump genes and *acrB* were the most solicited [67]. Camp et al. recently explored the efflux mechanisms involvement in an international collection of *E. coli* exhibiting an MDR phenotype [68]. By using MICs tests with and without the addition of the AcrAB-TolC efflux inhibitor 1-(1-naphthylmethyl)-piperazine (NMP) and by qRT-PCR, they identified in 50% of strains an efflux mechanism associated with antimicrobial resistance. They showed a significant overexpression of the AcrAB-TolC system in the 17 corresponding strains. Moreover, whole-genome sequencing indicated amino acid substitutions in AcrR, MarR, and SoxR genetic regulators.

#### 4.4.4. *E. cloacae*

Several teams reported the intervention of efflux pumps belonging to RND and MATE families in resistant *E. cloacae* stains [69]. The involvement of AcrAB, EefABC and OqxAB in the expel of fluoroquinolones, tetracycline and chloramphenicol is well-characterized in some clinical strains [46,69,70]. However, only a few publications have evaluated the efflux prevalence in clinical isolates collections or attempted to follow the evolution of efflux resistance during recent decades. Rosa et al. studied 25 *E. cloacae* carbapenem resistant strains, derived from the same hospital and likely of clonal origin. They found in most strains an overexpression of AcrAB by SDS-PAGE assays associated with carbapenamase KPC expression. They concluded that efflux may be associated with carbapenemase production to confer a high level of resistance to meropenem and imipenem. For them, efflux pumps enhanced carbapenemases action and completed the MDR phenotype [71].

Telke et al. studied two strains of *E. cloacae* and *E. asburiae* which were isolated from stools and presented a colistin hetero-resistant phenotype. They demonstrated that colistin hetero-resistance was due to the over expression of AcrAB upregulated by the *soxRS* regulation [72].

Hang Liu et al. investigated the involvement of efflux mechanisms in 140 *E. cloacae* tigecycline resistant strains isolated from various samples between 2014 and 2017. Efflux pump inhibitory assays with PAβN and the quantification of efflux pump genes expression by qRT-PCR showed that *acrAB* and *oqxAB* were upregulated. Antibiotics MICs with PAβN were reduced in 60% of strains; *acrAB* were upregulated in 78% and *oqxAB* in 28.6% of the strains. The genetic regulator RamA was expressed in 57% of strains [73].

### 4.5. Burkholderia cepacia, thailandensis and pseudomallei

Several members of the genus *Burkholderia* are dangerous pathogens. Infections caused by these bacteria are difficult to treat because of their significant level of antibiotic resistance [74]. Although efflux pumps are described in several *Burkholderia* species, only specific studies are carried out in *B. cepacia* complex and *B. pseudomallei*, in which they confer resistance to aminoglycosides, chloramphenicol, fluoroquinolones, and tetracyclines as tigecycline [75].

In the *B. cepacia* complex, several efflux pumps (MexA, MexC, MexE, and MexX) are involved in MDR [75,76]. Gautam et al. investigated the relation between the expression of efflux pumps, outer membrane porin OprD, and the β-lactamase AmpC, with antimicrobial susceptibility among 44 clinical isolates of *B. cepacia* complex [77]. They found that the reduced susceptibility to chloramphenicol was correlated with the overexpression of (*mexC*, *mexE*, *and mexX*) in the majority (>95%) of the isolates. Increased *mexA* expression showed a significant association with reduced susceptibility to β-lactam and co-trimoxazole. Increased *mexC* and/or *mexX* was associated with a reduced susceptibility to meropenem. Finally, a reduced susceptibility to ceftazidime and levofloxacin was associated with *mexE* and *mexX* expression, respectively [77].

*B. thailandensis* is closely related to *B. pseudomallei*, but non-pathogenic to humans. In *B. thailandensis*, three efflux complexes, AmrAB-OprA, BpeEF-OprC, and BpeAB-OprB are expressed and provide protection against multiple antibiotics, including polymyxins [78]. Authors show that the inactivation of AmrAB-OprA or BpeAB-OprB potentiates the antibacterial activities of several antibiotics. BpeF expels chloramphenicol, trimethoprim/sulfamethoxazole, and quinolones. Biot et al. demonstrated that, in doxycycline-selected variants, two overexpressed efflux complexes co-exist depending on the selected doxycycline concentration: AmrAB-OprA and BpeEF-OprC [78]. BpeEF-OprC likely took over from AmrAB-OprA at high resistance levels to doxycycline in strains, in which AmrB was downregulated. The expression of the BpeAB-OprB was reduced in doxycycline-resistant variants, whereas the AmrAB-OprA was overexpressed. Interestingly, BpeAB-OprB, could substitute to one of the present complexes when defective. In *B.thailandensis* an efflux pump was present, which belonged to the MSF group, in strains such as EmrB of *E. coli* [79,80]. In *B.thailandensis,* trimethoprim and tetracycline were expelled by this pump and the *emrB* gene was upregulated following the addition of gentamicin, due to the repression of OstR, a member of the MarR family repressor.

*B. pseudomallei*, the agent of melioidosis, is naturally resistant to many antibiotics [81,82,83]. Three RND complexes (AmrAB-OprA, BpeAB-OprB and BpeEF-OprC) are characterized in *B. pseudomallei*, two of which confer either intrinsic or acquired resistance to several antibiotic families, using trimethoprim+sulfamethoxazole [81]. It is shown that AmrAB-OprA in *B. pseudomallei* is associated with aminoglycoside and macrolide resistance. The meropenem is the gold standard treatment option. A clinical collection of *B. pseudomallei* isolates, including paired isolates which evolved during treatment, with reduced meropenem susceptibility were studied. Among them, 11 strains developed a decreased susceptibility toward meropenem during treatment. It was identified that, in such strains, multiple mutations affecting RND efflux pump regulators, with the concomitant overexpression of their corresponding pumps, rended strains refractory to treatment. In the expression of the three previously identified RND efflux pumps, as well as four other uncharacterized pumps, clinical isolates were found to be widespread [79]. In 45 of 50 isolates (90%), mRNA was detected for at least one of the seven RND pumps and among them, 41 (82%) expressed multiple pumps, nine expressing all the seven pumps tested. There was no striking correlation between RND efflux pump expression and clinically significant antibiotic resistance, however, RND pumps played important roles in the protection against toxic compounds and resistance.

### 4.6. Stenotrophomonas maltophilia

*Stenotrophomonas maltophilia* is a soil-borne bacterium often isolated in nosocomial infections due to its natural resistance to various antibiotics, e.g., carbapenems, and its ability to colonize cancer in immunocompromised or cystic fibrosis patients [84,85,86]. The species is characterized by a great genetic diversity confirmed by various typing methods and the multilocus sequence typing method [87,88,89,90]. Efflux is a major mechanism of adaptation in this species and is involved in intrinsic resistance. The presence of many efflux pumps: 8 RND (SmeABC, SmeDEF, SmeGH, SmeIJK, SmeMN-TolC, SmeOP-TolC, SmeVWX, SmeYZ), and 2 ABC (SmrA, macABC), as well as two MFS transporters encoding genes are described by whole-genome strains [84,91]. Youenov et al. identified 3 MATE, 3 SMR, 4 MFS and 4 ABC pumps in the three clinical strains sequenced [86]. The pumps of Sme series were confirmed as involved in intrinsic as-acquired resistance described in clinical MDR strains [84,92,93]. SmeABC was involved in resistance to β-lactams, aminoglycosides, trimethoprim/sulfamethoxazole, and quinolones; SmeDEF in resistance to chloramphenicol, ceftazidime, tetracycline, fluoroquinolones and triclosan; SmeJK in resistance to aminoglycosides, tetracycline, and ciprofloxacin; SmeOP-TolC in resistance to a low susceptibility of aminoglycosides, nalidixic acid, doxycycline; and, finally, SmeVWX as SmeYZ in resistance to aminoglycosides [84,88,92].

In clinical isolates, the three RND efflux systems, SmeABC, DEF and VWX are the most identified [92,93,94,95,96]. Real-time PCR analysis shows the overexpression of *smeB* in 21 (63.6 %) and *smeF* in 19 (57.5%) of 33 clinical isolates [96]. Fifteen (45.4%) isolates overexpressed both *smeB* and *smeF*. Interestingly, *S. maltophilia* is the only known bacterium in which quinolone-resistance is not associated with mutations in the genes encoding bacterial topoisomerases [88]. SmeDEF and SmeVWX are responsible for a part of the fluoroquinolone’s resistance. As illustrated, in 31 clinical *S. maltophilia* isolates presenting an MIC range to ciprofloxacin between 0.5 and >32 μg/mL, there were 11 overexpressed SmeDEF and two overexpressed SmeVWX [95]. The strains overexpressing SmeVWX presented changes at the Gly266 position of SmeRv, the repressor of SmeVWX [95]. In acquired resistance, the overexpression of the SmeDEF and SmeVWX efflux systems in clinical strains are frequently related to mutations in the regulators SmeT and SmeRv, respectively [97]. SmeT appears to play a central role in the adaptive resistance to quinolones and other expelled antibiotics, like tetracycline, chloramphenicol, erythromycin, and aminoglycosides. Resistance from a mutation emerges during a short course of ciprofloxacin, and quinolone monotherapy is not recommended for *S. maltophilia* bacteremia [98].

### 4.7. Pseudomonas aeruginosa

In *P. aeruginosa*, several multidrug transporters belonging to the RND family, such as MexAB, MexCD, MexEF, MexGHI, MexJK, MexXY and MexVW are reported to expel various antibiotics and biocides [99]. The most prevalent efflux complexes are: (i) MexAB-OprM, that confers resistance against tetracycline, chloramphenicol, quinolones, trimethoprim, and most β-lactams; (ii) MexXY, that contributes to the resistance against aminoglycosides, quinolones, tetracycline, erythromycin, and (iii) MexCD-OprJ for which the upregulation correlates with an increased resistance to ciprofloxacin, cefepime, and chloramphenicol [100,101].

The regulation of the major efflux system MexAB-OprM of *P. aeruginosa* is quite complex. Mutations in at least three different regulatory proteins genes from the TetR family of repressors (*mexR*, *nalC*, and *nalD*) can provide for the increased expression of MexAB-OprM [102]. It must be noted that the pump also plays a role in the production of a few virulence factors [102]. Sobel et al. demonstrated that MexAB-OprM can be overexpressed without mutations in *mexR* or *nalC,* but mutations in *nalD* are reported in some clinical strains [102]. Elsewhere, NalD is an attractive target for developing compounds to dysregulate the major pumps expression. In 2004, a French multicentre study investigated mechanisms of β-lactam resistance in 450 non-redundant strains of *P. aeruginosa* obtained from 15 French university hospitals [101]. The overproduction of the MexAB-OprM efflux system was present in 22.3% of the strains. In 2007, an Algerian study on 199 strains demonstrated an overproduction of MexAB-OprM in 24% of strains [103]. Currently, depending on the studies, overexpression of *mexA* and *mexB* measures between 50 to 88% in clinical *P. aeruginosa* strains [104,105]. A study investigated 147 *P. aeruginosa* isolates from 89 clinical samples from various hospital countries (Australia, USA, and the Netherlands), 20 veterinary and 38 wastewater origins collected from 2012 to 2017 [106]. Among them, 15 isolates showing at least a fourfold reduction in MIC in the presence of PAβN were selected for qRT-PCR. Results indicated an overexpression of the MexA pump in all tested isolates. The highest level of overexpression (sixfold) of MexA was observed in a clinical isolate from a cystic fibrosis patient. MexAB-OprM was investigated for its role in conferring meropenem resistance and the effect of the single-dose exposure of meropenem on the transcription level of *mexA* [107]. Out of 83 meropenem non-susceptible isolates, 38 exhibited efflux pump activity against meropenem; 22 of these overexpressed MexAB-OprM. In most cases, meropenem could increase *oprM* and *oprN* mRNA levels [107].

In a collection of 110 French *P. aeruginosa* strains, Jeannot et al. reported that 3.7% of strains were *nfxB* mutants and exhibited moderate resistance [108]. The alteration of the *nfxB* gene, which coded for the repressor of the *mexCD-oprJ* operon, led to the overproduction of MexCD-OprJ and to fluoroquinolones, macrolides, cefpirome and cefepime resistance [108]. However, some “*nfxB* mutants” are more susceptible than wild-type strains to aminoglycosides, aztreonam, and imipenem. In contrast to MexAB-OprM and MexXY-OprM, the MexCD-OprJ system does not contribute to the natural resistance of *P. aeruginosa*. This phenotype is appreciable, despite high rates of gain-of-efflux mutants (MexAB-OprM, MexXY-OprM) in France. Indeed, very few *nfxB* strains are identified in the clinical setting, probably in connection with the fact that these bacteria present an impaired fitness. Yet, MexCD mutants are quite significant in cystic fibrosis patients and the pump is known to potentiate the effect of mutations in target genes as *gyrA* [108].

Regarding the MexEF-OprN, a study concerning 221 multidrug-resistant strains of *P. aeruginosa* with reduced susceptibility to ciprofloxacin, reported that 43 (19.5%) overproduced the pump [109]. The *mexEF-oprN* operon was controlled by an adjacent gene *mexT*, a LysR-type activator, which itself was regulated by *mexS,* a gene involved in the detoxification of some MexT-activating molecules. Among the 43 strains, only three (13.6%) contained a disrupted *mexS* gene and nine presented a *mexS* mutation, whose inactivation is known to activate the *mexEF-oprN* operon through MexT. Single-point mutations in *mexS* (40.9% of resistant strains) represent a significant cause of MexEF-OprN upregulation. Another study investigated the resistance mechanisms to fluoroquinolones of 85 non-cystic fibrosis *P. aeruginosa* exhibiting a reduced susceptibility to ciprofloxacin [110]. Authors found an upregulation of MexAB-OprM (36% of isolates), MexXY/OprM (46% of isolates) and MexEF-OprN efflux pump (12% of isolates). An analysis of the 10 MexEF-OprN overproducers indicated the presence of various mutations in the *mexT* (two isolates) or *mexS* (five isolates). Importantly, MexEF-OprN represented a key mechanism by which *P. aeruginosa* could acquire higher resistance levels to fluoroquinolones and was underestimated.

MexXY efflux mutants are frequent and described as selected by aminoglycosides, alone or in combination with fluoroquinolones [111]. Moreover, resistance to cefepim and/or ceftazidime is mostly due to the stable overproduction of MexXY that is demonstrated in 32 clinical strains [111]. Thirty three percent of strains resistant to cefepim overexpressed the gene *mexY.* Moreover, the simultaneous overexpression of MexXY and MexAB-OprM in clinical isolates results in a twofold increase in cefepime MIC, compared with single MexXY production [112]. Mutations inside or outside of the regulatory gene, *mexZ* (*agrZ* or *agrW* mutants, respectively), or the TCS system, *armgR/S,* which controls the expression of the operon, *mexXY,* are involved in the pump overexpression [113]. The simultaneous overexpression of MexAB-OprM, MexEF, and MexXY is not exceptional [112]. Additionally, a single clinical strain overexpressed the three RND genes tested in this work. The same authors investigated the resistance mechanisms to β-lactams, aminoglycosides, and the fluoroquinolones of 120 bacteremic strains of *Pseudomonas aeruginosa* [114]. They found that 11 and 36% of the isolates appeared to overproduce the MexAB-OprM and MexXY-OprM efflux systems, respectively. Del Barrio Toffino et al. studied 150 toto-resistant clinical isolates, recovered in 2015 from different sites from nine hospitals in Spain [115]. Via the analysis of the efflux pump gene expression coupled to the sequencing of their regulatory components, they reported that frequent mutations of *mexZ* generated an overexpression of MexXY-OprM. Mutations were detected in most (73%) of the strains analyzed. Moreover, 25% of the strains, were *mexR* (*nalB*), *nalC*, or *nalD* variants and were also overexpressed in the MexAB-OprM pump. *mexT* mutations, associated with MexEF-OprN overexpression and OprD downregulation, were detected in a few strains and two isolates showed MexCD-OprJ overexpression due to *nfxB* mutations, contributing to ciprofloxacin resistance. Additionally, several sequence variations in unique residues were detected in the efflux pump components. Despite this, many of the studied isolates overexpressed several efflux pumps; the major regulator, *mexZ,* controlling MexXY expression was very often mutated (70.5%), highlighting the relevance of MexXY overexpression as reported in other studies [116]. Among 57 unrelated strains from non-cystic fibrosis patients, 44 (77.2%), named *agrZ* mutants presented mutations inactivating the local repressor gene, *mexZ* [117]. These mutations, were located, in the dimerization domain, the DNA-binding domain or the affected amino acid positions of the TetR-like regulators. Five strains (8.8%), harbored single amino acid variations in ParRS, a two-component system known to positively control *mexXY* expression. Some studies demonstrated the involvement of other genetic alterations in the upregulation of MexXY in clinical strains [118]. For example, mutations in the elongation factor G (EF-G1A) potentiate aminoglycoside resistance in MexXY pump mutants [117]. In conclusion, clinical strains of *P. aeruginosa* exploit three distinct regulatory pathways, mutations in the local repressor, MexZ, in the MexZ antirepressor, ArmZ, and/or in the two-component regulatory system, ParRS, that contribute to overproduce the MexXY-OprM. The use or combination of these ways explain the high prevalence of MexXY-OprM mutants in the clinical samples [115]. Very recently, an Italian study investigated the prevalence of aminoglycoside resistance in 147 *P. aeruginosa* strains isolated from respiratory samples from Cystic Fibrosis patients. Of these, 78 (53%) were resistant to at least one aminoglycoside and overexpressed the MexXY-OprM system [119].

From 122 isolates obtained from three hospitals in Iran, most of strains expressed *mexB* (69%), *mexC* (28.7%), *mexE* (43.4%), and *mexY* (74.6%), suggesting that *mexB* and *mexY* were highly expressed in ICU wards [120].

Finally, several studies demonstrated the involvement of two or three different pumps in the resistance of clinical strains: 33 clinical strains of *P. aeruginosa* from French hospitals, resistant to ciprofloxacin and 30 non-clinical strains originating from the hospital waterborne environment, were collected during a 5-month period and included in the study [121]. The overexpression of *mexB, mexF* and *mexY* was detected in 27, 12, and 45% of the clinical strains, respectively. In the 30 non-clinical strains, no overexpression could be found for all genes studied. In both clinical and environmental strains, a positive correlation was found between the expressions of *oprD, mexB* and *mexF*. However, in clinical strains, no statistically significant link could be found between the extent of reduction in ciprofloxacin MICs in the presence of PAβN and the expression of any of the three efflux genes studied [121]. In Iran, 154 *P. aeruginosa* strains recovered from the clinical burn wound and resistant to ciprofloxacin, were studied [122]. The *mexA, mexC* and *mexE* genes were recovered in 95.4% isolates simultaneously, but according to the phenotypic investigation of the efflux pump, with MICs studies with CCCP, half of the genes showed an overexpression of multidrug efflux pumps. Furthermore, 59 *P. aeruginosa* clinical isolates were obtained from 57 patients and the efflux pump inhibitor PAβN was used to determine the contribution of the RND pumps in the resistance to carbapenems and ciprofloxacin [123]. Authors found that 17 strains showed a decrease in ciprofloxacin associated with efflux mechanisms. A total of 634 *P. aeruginosa* clinical isolates were collected from various clinical specimens from patients treated by carbapenems [124]. Most of the isolates displayed the gene over-expression of *mexCD-oprJ* (75%) followed by *mexXY-oprM* (62%), while *mexAB-oprM* and *mexEF-oprN* genes were overexpressed in 21.8% and 18.7% of the isolates, respectively. A correlation was found between the MexXY and MexCDJ efflux pump expression and meropenem, or imipenem resistance in more than 60% of resistant strains. Three of the isolates showed an overexpression of the four tested efflux pumps [124]. A Polish study, investigated the role of the efflux pump in 73 cefepime and/or ceftazidime-resistant strains isolated between 2004 and 2011 by using the inhibitor, PAβN. The restoration of susceptibility to cefepime and/or ceftazidime in the majority of ESβL-positive *P*. *aeruginosa* strains with low and moderate levels of resistance to cefepime indicated that RND efflux pumps had a significative impact on susceptibility to β-lactams [14].

### 4.8. Acinetobacter baumannii

*Acinetobacterbaumannii* is a major nosocomial pathogen associated with a high mortality in immunocompromised patients, and the emergence of multidrug-resistant strains has increasingly been reported [125,126]. Efflux mechanisms, in the species, are suspected to contribute to intrinsic resistance to broad spectrum antibiotics since several years [127]. Three RND efflux systems, AdeABC, AdeFGH, AdeIJK and AdeXYZ are characterized in *Acinetobacter* species [126,127,128]. AdeABC, the most prevalent efflux system, is associated with resistance to β-lactams, such as: ticarcillin, cephalosporins, aztreonam, carbapenem, tetracyclines, tigecycline, aminosides, fluoroquinolones, lincosamides, rifampin, chloramphenicol, cotrimoxazole, novobiocin, and fusidic acid [127,129]. The over-expression of AdeABC is controlled by the AdeRS two-component system, and mutations in the *adeRS* genes efficiently contribute to multiresistance [126]. *AdeFGH*-overexpressing mutants are resistant to fluoroquinolones, chloramphenicol, trimethoprim, and clindamycin and have a decreased susceptibility to tetracyclines, tigecycline, and sulfamethoxazole without affecting β-lactams and aminoglycosides phenotypes [130]. AdeIJK is responsible for the natural resistance of the species and associated with resistance to cyclins, third generation cephalosporins, sulfamides, fluoroquinolones, and cyclines. However, its overexpression above a certain threshold is toxic for the host and its contribution to the acquired resistance is limited [126,131,132]. The expression of AdeFGH and AdeIJK is controlled by AdeL, a LysR-type transcriptional regulator and AdeN, a TetR-like transcriptional regulator, respectively [133]. Non-RND efflux systems, such as MFS pumps, CraA, AmvA, TetA, TetB, CmlA, FloR, or MATE pumps, similar to AbeM, have narrow-spectrum efflux profiles and are mainly encoded by mobile genetic elements [125,126]. AmvA extrudes mainly dyes, disinfectants, and detergents [134]. Erythromycin is the only antibiotic for which the activity is significantly increased, with a fourfold decrease in the MIC when the structural gene is inactivated. CraA is homologous to the MdfA efflux pump of *E. coli*, which extrudes only chloramphenicol [135]. The inactivation of CraA in *A. baumannii* results in a 128-fold decrease in chloramphenicol resistance. The system is found in all 82 *A. baumannii* strains tested and contributes to the intrinsic resistance to chloramphenicol. Several Tet efflux pumps conferring tetracycline resistance are acquired by *A. baumannii* clinical isolates [136]. TetA and TetB are the most prevalent, with TetA conferring resistance to tetracycline only and TetB also expelling minocycline [137]. CmlA and FloR efflux systems confer resistance to phenicols and are found in the AbaR1 resistance island [138]. AbeM, belonging to the MATE family, extrudes aminoglycosides, fluoroquinolones, chloramphenicol, trimethoprim, ethidium bromide, and dyes [139]. Regarding the SMR family, AbeS, a chromosomally efflux pump displaying homology with the EmrE system of *E. coli*, has been recently characterized in an *A. baumannii* MDR clinical isolate and is responsible for resistance to erythromycin, fluoroquinolones, and chloramphenicol [136,140,141]. Several studies have identified the involvement of efflux in MDR phenotypes of collection of clinical strains. Lari et al. observed that 14 of the 16 efflux-positive isolates with full resistance to ciprofloxacin, overexpressed *adeB* [142]. In 95 *A. baumannii* clinical strains, phenotypic assays with PAβN demonstrated that 40% of the isolates expressed a sensitive PAβN-efflux mechanism, mainly against tigecycline [143]. The *adeA* gene was detected in more than 73% of clinical strains, and the expression of pump genes, *adeB, adeG* and *adeJ* was observed in some of these by qRT-PCR [144]. Bratu et al. reported that in the absence of cephalosporinase activity, the AdeABC efflux system was responsible for the reduced susceptibility to cefepime [145]. Sixty-eight ciprofloxacin-resistant clinical isolates were investigated for their efflux pumps genes profile [146]. They all had an overexpression of *adeB* and, in 73% of them, mutations were characterized in the regulatory system of the pump AdeRS. In particular, mutations within the two-component AdeRS correlated with the high tigecycline MICs in southern European countries. Several substitutions in *adeS* were found to be responsible for *adeABC* overexpression [145]. Yoon et al. screened 14 MDR non-redundant clinical isolates for the presence and overexpression of the three Ade efflux systems and analyzed the sequences of the regulators, AdeRS and AdeL [147]. The gene, *adeB,* was detected in 13 of 14 isolates; *adeG* and the intrinsic *adeJ* gene were detected in all strains. The significant overexpression of *adeB* was observed in 10 strains, whereas only seven had moderately increased levels of expression of AdeFGH; however, nonoverexpressed AdeIJK pumps. Thirteen strains displayed a reduced susceptibility to tigecycline, but there was no correlation between tigecycline MICs and the levels of AdeABC expression. No mutations were found in the AdeL regulator of the nine strains expressing AdeFGH. In contrast, functional mutations were found in the conserved domains of AdeRS in all the strains that overexpressed AdeABC, with two mutational hot spots: one located in AdeS and the other in the DNA-binding domain of AdeR compatible with horizontal gene transfer. The authors confirmed the high incidence of AdeABC efflux pump overexpression in MDR strains because of a panel of single mutations in the corresponding two-component regulatory system. Among 47 *A. baumannii* strains from the blood of septicemic neonates from an Indian hospital, efflux-based fluoroquinolones resistance was found in 65% of strains with at least two different active pumps; AdeAB and AdeIJ, or AdeFG in 38% of strains [148]. Amino acid changes in the regulators (AdeRS/AdeN/AdeL), either as single or multiple substitutions, were responsible for the overexpression of the pumps and detected simultaneously among 64% of resistant strains. The AdeFG efflux pump was specifically associated with fluoroquinolones resistance in 200 clinical strains from Iran [131]. It was found in 90% of clinical strains and was responsible for high levels of resistance to chloramphenicol, clindamycin, fluoroquinolones, and trimethoprim [131]. From a total 313 isolates, 113 tigecycline-resistant isolates were analyzed [149]. The most frequent mechanism associated with tigecycline resistance was the disruption of the repressor, AdeN, to the AdeIJK system, by IS elements or nucleotide deletions causing premature stop codons. The overexpression of *adeB, adeJ, adeM and adeG* genes was detected among 70%, 53%, 30% and 23% of 30 *A. baumannii* strains, respectively, which showed at least a fourfold decrease in MICs for FQs in presence of efflux inhibitors [150]. The relative expression level of *adeB* was highest (2.2- to 34-fold) among all the pumps tested. This suggested that, although the involvement of *adeB* was most described in FQ resistance, the overexpression of other pumps cannot be overlooked in the future. Since the AdeIJK pump was intrinsic to *A. baumannii*, it was detected among large numbers of strains, but, in comparison to *AdeB*, the relative fold change of *AdeJ* production was low, supporting the fact that the overexpression of AdeIJK was phenotypically toxic to *A. baumannii* [132]. In five isolates, the overexpression of AdeABC was not detected either in AdeRS or mutations within AdeRS. In these isolates, an association of overexpression of other pumps (*adeJ* or *adeG* or *abeM*) with elevated FQ MICs was observed. In this study, at least two different pumps were overexpressed simultaneously among 38% of fluoroquinolone-resistant strains [132]. One hundred *A. baumannii* clinical isolates were assayed for the inhibitory effect of reserpine and CCCP on the antimicrobial susceptibility and expression of 4 RND-type multidrug efflux systems, including AdeABC, AdeDE, AdeIJK, and AdeFGH, using RT-PCR [151]. Ten *A. baumannii* isolates expressing AdeABC, AdeIJK, or AdeFGH were randomly selected for the determination of the transcription level and regulatory mutations. The reserpine and CCCP experiment showed that the mulidrug resistance phenotype in most *A. baumannii* isolates was associated with the presence of active efflux pumps under these conditions. Most isolates expressed at least one of the RND-type efflux pumps tested (97%). AdeIJK expression was most common (97%) as none of the isolates produced AdeDE; 52% of isolates simultaneously produced up to three RND-type efflux systems (AdeABC, AdeFGH, and AdeIJK). Importantly, authors couldn’t correlate the expression of RND-type efflux pumps and the MDR phenotype [151].

## 5. Conclusions, Therapeutic Solutions to Efflux

Clinically used antibiotics are well-recognized and actively expelled by efflux pumps which are expressed in clinical resistant isolates [1,2,4]. The appropriate methods are now available (e.g., fluorimetry, mass spectrometry) to monitor the intra-bacterial accumulation of antibiotics and these methods can be used to measure the effectiveness of countermeasure developed to block efflux activity, and to compare these measurements with the expected increase in bacterial susceptibility [1]. However, this approach is not widely used and only the direct effect of the combination of antibiotic + adjuvant (by fractional inhibitory concentration, FIC, or other calculations [154]) on bacterial susceptibility is currently used, generating some uncertainties in the characterization of the mode of action for a putative adjuvant. Regarding the possible ways to circumvent this efflux mechanism and restore the antibiotic action of resistant bacteria, numerous publications described the collection of molecules that collapse the efflux in Gram-negative bacteria, and synthetic or natural compounds [6,11,155,156].

Impairing the efflux activity can be achieved by using different approaches: (i) decreasing the affinity of efflux system for the antibiotic, (ii) increasing the antibiotic penetration rate, (iii) saturating the efflux capability by competitors that mimic the substrate, (iv) dissipating the energy source of expel, (v) blocking the exit channel, and (vi) downregulating or destabilizing the efflux system.

### 5.1. Working on Molecule Profile

An interesting strategy regarding the molecule itself was recently opened with the study reporting the susceptibility of antibiotics belonging to the fluoroquinolone (FQ) family to efflux pump [3]. In this paper, the authors have used the Structure Concentration Intracellular Activity Relationship (SICAR) indexes [3,12] to illustrate the penetration and the accumulation rate of various fluoroquinolones in isogenic stains expressing the various levels of the AcrAB pump. This ranking using the SICAR_EFF_ allows for the identity of some chemical chains that are noticeably involved in expelling efficacy and recognized by AcrB sites [3]. Importantly, this study paves the way for future rational pharmacomodulations of the antibiotic class to decrease its sensitivity to efflux and to consequently restore the internal concentration necessary to kill the bacteria.

Alternatively, it has been reported that when the antibiotic concentration in the medium increases, the total efficacy of efflux decreases [10]. This suggest that the level of resistance conferred by efflux depends on the antibiotic permeation rate across the bacterial membrane and its intrabacterial accumulation [1,7]. This “saturation” of efflux capability can be also reached by using appropriate lures that mimic the antibiotic structure and induce a competition for the efflux sites inside the pump.

### 5.2. Working on Pump Activity-Structure

Since RND efflux pumps require the inner membrane energy (proton motive force) as the driving force during drug transport, an attractive idea was to dry up the energy source and collapse the energy–transport coupling [2]. Unfortunately, carbonyl cyanide *m*-chlorophenylhydrazone is more efficient and widely used as an energy dissipator, and it is also a strong poison which cannot be used in clinic.

Regarding alternative methods, several molecules are described as restoring antibiotic susceptibility by destabilizing the membrane or inducing some hindrances for the access of AcrB affinity pockets that recognize and capture the antibiotics [4,156,157]. The feasibility of blocking the TolC channel, carried out in vitro, could be an attractive way to restore the antibiotic concentration inside the bacterial cells with many advantages (specificity, target located at the outer membrane surface), but the proof of this idea remains to be demonstrated on intact resistant bacteria [152]. Finally, the downregulation of efflux pump genes, by blocking the role of activators, such as MarA or RamA, seeFms to be difficult to exploit due to the redundancy of regulators and the presence of genetic overlaps controlling the expression of efflux systems.

### 5.3. Assays and Clinic Aspects of Efflux Inhibitors

At this moment, several β-lactamase inhibitors are routinely used in combination with β-lactams to combat and eradicate β-lactamase producers. In contrast, no efflux blockers passed the different steps to be validated and accepted for clinical use. Different aspects are involved in the lack of development of efflux blockers and are discussed in recent publications: chemical structures different from usual antibiotics, combination aspects. and rules in regulatory affairs (patents and licensing, etc.) [155,158,159].

Today, the clear identification and characterization of the efflux role in resistant clinical isolates in the research laboratory using recently developed methods (mass spectrometry, fluorimetry, and rate-killing assays, etc.), and its contribution to the spreading of the resistance phenotype in hospital wards clearly demonstrate the need for a robust diagnostic of efflux strains and theie associations with other membrane-associated mechanisms of resistance that contribute to MDR emergence. In addition, the molecular dissection of expel transport, and the dialogue between the antibiotic and bacterial transporter (pump and channel) are essential to circumvent the efflux capability and restore the antibiotic concentration on the target inside the bacterial cell.

## Figures and Tables

**Table 1:** The various pumps identified in Gram-negative clinical strains as involved in antibiotic resistance, N.D; (Not Determined).

Species	Efflux Pump Family	Characterized Efflux System Involved in Resistance of Clinical Strains	Identified Regulator(s)	Refs
*Haemophilus influenzae*	RND	AcrAB homolog	AcrR homolog	[19,22]
*Helicobacter pylori*	RND	HefABC, HefDEF, HefGHI	N.D.	[23,26]
*Campylobacter jejuni*	RND	CmeABC, CmeDEF, RE-CmeABC	CmeR	[30,33,34]
*Klebsiella pneumoniae*	RND	AcrAB, OqxAB, KexD, AcrCD, MdtABC	MarA, RamA, SoxS, Rob, RarA, AcrR, H-NS, CpxAR, SdiA, RamR	[7,40,44,54,55]
	SMR	KpnEF	N.D.	[23,53,54,55]
	MATE	KdeA	N.D.	[54,55]
	ABC	MacAB	N.D.	[55]
	MFS	EmrAB	N.D.	[53]
*Klebsiella aerogenes*	RND	AcrAB, AcrCD, MdtABC, OqxAB, EefABC, RosAB	MarA, RamA, SoxS, Rob, RarA, AcrR, H-NS, CpxAR, SdiA, RamR	[7,46,56,57,58,59,60]
	ABC	MacAB	N.D.	[46,56]
	MFS	EmrAB	N.D.	[46,56]
*Escherichia coli*	RND	AcrAB, AcrCD, AcrEF, MdtABC, MdtEF, YihV	MarA, SoxS, Rob, RarA, AcrR, H-NS, CpxAR, SdiA, BaeSR, MarR, SoxR	[7,64,65]
	MFS	MdfA	N.D.	[66]
	ABC	YdcV, YdcU, YdcS, YdcT, CysP	N.D.	
*Enterobacter cloacae*	RND	AcrAB, EefABC, OqxAB	MarA, RamA, SoxS, Rob, RarA, AcrR, H-NS, CpxAR, SdiA, SoxRS, RamR	[7,46,69,70]
*Burkholderia cepacia*	RND	MexAB, MexCD, MexEF, MexXY	LysR family, Tet-R type regulator, MerR-type regulator	[75,76,152]
*Burkholderia thailandensis*	RND	AmrAB, BpeEF, BpeAB,	OstR	[78,80]
	MFS	EmrAB	N.D.	[79]
*Burkholderia pseudomallei*	RND	AmrAB, BpeEF, BpeAB	N.D.	[79,81]
*Stenotrophomonas maltophilia*	RND	SmeABC, SmeDEF, SmeGH, SmeIJK, SmeMN, SmeOP, SmeVWX, SmeYZ	SmeSR, SmeRySy, Tet-R type regulator, SmeT, SmeRv	[84,92,153]
	ABC	SmrA, macABC	N.D.	[84,86,91]
*Pseudomonas aeruginosa*	RND	MexAB, MexCD, MexXY, MexEF, MexGHI, MexJK, MexVW	MexR, NfxB, MexT, MexZ, MexL, NalD, NalC, ParRS, ArmZ, ArmgR/S, MexS	[99,100,101,102,153]
*Acinetobacter baumannii*	RND	AdeABC, AdeFGH, AdeIJK, AdeXYZ, AdeDE	AdeRS, AdeL, AdeN	[126,127,128,133,151]
	MFS	CraA, AmvA, TetA, TetB, CmlA, FloR	N.D.	[125,126]
	MATE	AbeM	N.D.	[125,126,139]
	SMR	AbeS	N.D.	[136,140,141]

## Data Availability

Not applicable.
